# An Investigation of Descending Pain Modulation in Women With Provoked Vestibulodynia (PVD): Alterations of Spinal Cord and Brainstem Connectivity

**DOI:** 10.3389/fpain.2021.682483

**Published:** 2021-08-12

**Authors:** Lindsey R. Yessick, Caroline F. Pukall, Gabriela Ioachim, Susan M. Chamberlain, Patrick W. Stroman

**Affiliations:** ^1^Department of Psychology, Queen's University, Kingston, ON, Canada; ^2^Centre for Neuroscience Studies, Queen's University, Kingston, ON, Canada; ^3^Department of Obstetrics and Gynecology, Queen's University, Kingston, ON, Canada

**Keywords:** pain, provoked vestibulodynia, neuroimaging, brainstem, spinal cord, chronic pain

## Abstract

The most common subtype of vulvodynia (idiopathic chronic vulvar pain) is provoked vestibulodynia (PVD). Previous imaging studies have shown that women with vulvodynia exhibit increased neural activity in pain-related brain regions (e.g., the secondary somatosensory cortex, insula, dorsal midcingulate, posterior cingulate, and thalamus). However, despite the recognized role of the spinal cord/brainstem in pain modulation, no previous neuroimaging studies of vulvodynia have examined the spinal cord/brainstem. Sixteen women with PVD and sixteen matched Control women underwent a spinal cord/brainstem functional magnetic resonance imaging (fMRI) session consisting of five runs with no painful thermal stimuli (No Pain), interleaved randomly with five runs with calibrated, moderately painful heat stimulation (Pain). Functional connectivity was also assessed in periods before, during, and after, pain stimulation to investigate dynamic variations in pain processing throughout the stimulation paradigm. Functional connectivity in the brainstem and spinal cord for each group was examined using structural equation modeling (SEM) for both Pain and No Pain conditions. Significant connectivity differences during stimulation were identified between PVD and Control groups within pain modulatory regions. Comparisons of Pain and No Pain conditions identified a larger number of connections in the Control group than in the PVD group, both before and during stimulation. The results suggest that women with PVD exhibit altered pain processing and indicate an insufficient response of the pain modulation system. This study is the first to examine the spinal cord/brainstem functional connectivity in women with PVD, and it demonstrates altered connectivity related to pain modulation in the spinal cord/brainstem.

## Introduction

Provoked Vestibulodynia (PVD) is characterized by pain evoked by pressure to the vaginal entrance and is the most common subtype of vulvodynia (i.e., idiopathic chronic vulvar pain) (1). Studies have examined pain sensitization within and beyond the vestibular area via quantitative sensory testing (QST) and have demonstrated hypersensitivity in women with PVD as compared to Control women [e.g., (2–7)]. The possibility that central pain processing in the brain and spinal cord is modified in this condition has prompted a number of investigations into neuroanatomical and functional differences in women with vulvodynia (mostly PVD). These studies have provided evidence of altered pain processes in the form of greater gray matter density and altered microstructural organization in brain regions (8–10), augmented brain activity in pain processing regions in response to vulvar (11, 12) and thumb pressure (13), and alterations of sensorimotor, salience, and default mode network intrinsic connectivity (14). These results suggest that women with PVD may differ in peripheral and central processing of pain and sensory stimuli.

The spinal cord and brainstem contain key regions involved in pain processing and pain modulation, however, and studies of these regions in PVD are lacking. Without the brainstem and spinal cord, we can only obtain a partial picture of PVD pain. It is now recognized that BS structures, via descending facilitatory and inhibitory neurons, can enhance or suppress pain and sensory signaling from the SC to the brain (15). The perception of pain is influenced by descending pathways originating from the cortex that interconnect with BS structures and project to the dorsal horn of the SC to modulate ascending projections (16–18). Our understanding of these processes has already been enhanced by adaptations of fMRI methods to overcome the imaging challenges in BS/SC regions for fMRI (19–21). This fMRI method has been successfully used in pain-free participants under multiple pain manipulations (22, 23), as well as in populations with SC injury and fibromyalgia (19, 24). Using a paradigm involving the anticipation of a stimulus or absence of a stimulus, research has demonstrated continuous descending modulation in SC neurons (23). Despite the fundamental role of the BS/SC in descending modulation of pain and the availability of connectivity methods that infer directionality, no studies have examined neural processes in subcortical regions in women with PVD. The aim of this study was to examine descending modulation of pain within an a priori network model of subcortical BS regions and one SC region for women with PVD. We hypothesize that women with PVD will exhibit altered connectivity in regions known to be related to pain processing in the BS/SC cord, indicating diminished descending modulation.

## Methods

### Participants

Prospective participants were recruited through: (1) the Sexual Health Research Laboratory (SHRL) database, consisting of past participants who consented to being contacted for future research, (2) advertisements posted in the Kingston community including the university campus, (3) social media (e.g., Facebook) advertisements, (4) pamphlets in doctors' offices, and (5) emails to health care providers who may provide care for women with vulvodynia (e.g., urologists, gynecologists, pelvic health physical therapists). This study was approved by the Queen's University Health Sciences Research Ethics Board and informed consent was obtained from each participant.

**Figure 1:**
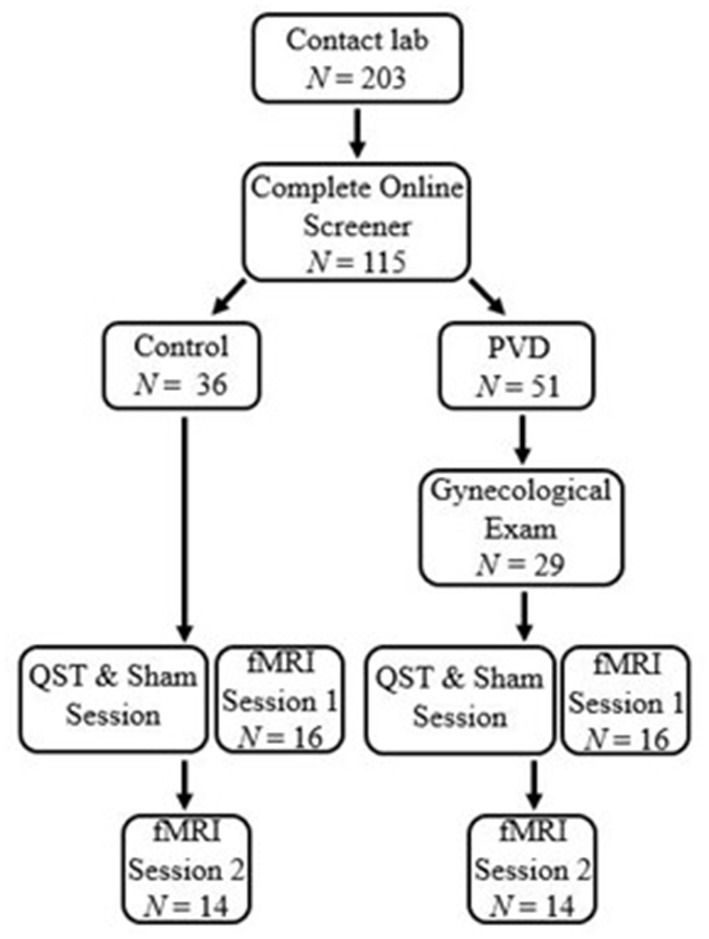
Participant flow chart. Participants first contacted the lab and completed an eligibility process. Women with PVD underwent a gynecological exam to confirm the presence of PVD symptoms. All participants underwent a thermal pain testing and Sham session to titrate paradigm heat stimulation and train participants in the paradigm. Immediately following the thermal pain testing and Sham session, participants were randomized to either a brain or BS/SC fMRI session, the first of which was completed at that time They then returned for a second session to complete their remaining fMRI session. This paper includes the results of the BS/SC portion of the study.

Participants were screened for eligibility either over the phone or online using Qualtrics survey software. Thirty-two women between 18 and 48 years of age were recruited for this study, 16 women in each group (PVD, Control). Significant effects have been found in previous studies that utilize similar analyses in populations with and without chronic pain conditions with similar sample sizes (19, 25). Demographic information for these participants can be found in Table 1. To reduce the influence of age-related changes or hormone use confounding the results, groups were matched on age (±5 years) and hormonal contraceptive use (yes or no) (26–28). Inclusion criteria for both groups included: older than 18 years of age or under 50; no magnetic resonance imaging (MRI) contraindications (e.g., metal implants); no major brain or spinal cord injury; no use of medications that substantially affect the central nervous system (e.g., antipsychotics); and not currently pregnant or breastfeeding. In addition, women with PVD had to report idiopathic, provoked pain to the vaginal entrance during sexual and non-sexual activities involving vaginal penetration. The results of the screening process and a gynecological examination (see below) formed the basis of inclusion or exclusion of women with PVD. For Control women, the primary inclusion criterion was the absence of self-reported chronic vulvar pain. Figure 1 illustrates the study procedure and the number of participants who completed each phase.

**Table 1:** Participant demographic information.

	**PVD sample**	**Control sample**	**Total sample**	***p*-value**
	***n* = 16**	***n =* 16**	***n* = 32**	
Age [*M* (*SD*)]	30.1 (9.8)	30.0 (9.7)	30.0 (9.6)	0.765
Sexual Orientation [*n* (%)]				0.101
Heterosexual	16 (100.0)	12 (75.0)	28 (87.5)	
Bisexual		2 (12.5)	2 (6.3)	
Same-sex attracted		1 (6.3)	1 (3.1)	
Not sure		1 (6.3)	1 (3.1)	
Relationship Status [*n* (%)]				0.744
Married	7 (43.8)	2 (12.5)	9 (15.6)	
Common-law	2 (12.5)	3 (18.8)	5 (15.6)	
Dating partner (regularly)	2 (12.5)	2 (12.5)	4 (12.5)	
Single (not dating)	1 (6.3)	2 (12.5)	3 (9.4)	
Casual sex (one partner)	1 (6.3)	1 (6.3)	2 (6.3)	
Casual sex (multiple partners)	1 (6.3)	2 (12.5)	3 (9.4)	
Dating partner (long distance)	1 (6.3)	2 (12.5)	3 (9.4)	
Living with partner	1 (6.3)	2 (12.5)	3 (9.4)	
Birthplace [*n* (%)]				0.476
Canada	13 (81.3)	12 (75.0)	25 (78.1)	
Latin/South America	2 (12.5)		2 (6.3)	
United States	1 (6.3)	1 (6.3)	2 (6.3)	
Europe		3 (18.8)	3 (3.1)	
Ethnicity [*n* (%)]				0.004
Canadian	13 (81.3)	8 (50.0)	21 (65.6)	
Latin	2 (12.5)		2 (6.3)	
North American Aboriginal	1 (6.3)		1 (3.1)	
European		4 (25.0)	4 (12.5)	
Asian		3 (18.8)	3 (9.4)	
Canadian Arabic		1 (6.3)	1 (3.1)	
Education [*n* (%)]				0.674
High school (complete)	2 (12.5)		2 (6.3)	
College/undergraduate degree (some)	3 (18.8)	5 (31.3)	8 (25.0)	
College/undergraduate degree (complete)	6 (37.5)	6 (37.5)	12 (37.5)	
Graduate/professional (some)	2 (12.5)	1 (6.3)	3 (9.4)	
Graduate/professional (complete)	3 (18.8)	4 (25.0)	7 (21.9)	
Income [*n* (%)]				1.00
$0–9,999	2 (12.5)	1 (6.3)	3 (9.4)	
$10,000–19,999	2 (12.5)	2 (12.5)	4 (12.5)	
$20,000–29,999	1 (6.3)	2 (12.5)	3 (9.4)	
$30,000–39,999	1 (6.3)	1 (6.3)	2 (6.3)	
$40,000–49,999		1 (6.3)	1 (3.1)	
$50,000–59,999	2 (12.5)	2 (12.5)	4 (12.5)	
$60,000+	6 (37.5)	6 (37.5)	12 (37.5)	
Decline Response	2 (12.5)	1 (6.3)	3 (9.4)	
History or Current Chronic Pain [*n* (%)]				
Back/Neck/Shoulder Pain	2 (12.5)			
Migraines	4 (25.0)			
Pain related to ovarian cysts	1 (6.3)			
Radiculopathy	1 (6.3)			
History of Depression and Anxiety [*M* (*SD*)]	*n = 15*	*n = 16*		
Beck Depression Inventory-II	13.0 (9.7)	10.1 (10.8)		0.444
Trait Subscale of the STAI	44.2 (12.3)	42.9 (11.3)		0.756

*Due to missing data, multiple responses, and rounding, not all percentages add up to 100*.

### Gynecological Exam

Vulvodynia is a diagnosis of exclusion (1). Therefore, the study gynecologist ruled out factors associated with vulvar pain (e.g., infections) in women believed to have PVD. The internal and external genitals and reproductive organs were visually and manually examined by the gynecologist. The gynecologist also performed the cotton-swab test of the external genitals (i.e., labia majora and minora, vestibule, perineum)—the main gynecological test for the diagnosis of PVD (29). The cotton-swab test involved the palpation of the labia majora, inner labia minora, midline areas, and six randomly ordered sites at the vestibule (e.g., 1 o'clock, 4 o'clock, 6 o'clock, etc.). Participants were asked to rate the intensity of the pain on a numerical rating scale ranging from 0 (no pain at all) to 10 (worst pain ever felt) after each palpation. In order to be included in the PVD group, a mean average pain intensity rating of at least 1/10 during the cotton-swab test of the vestibule was required. However, all participants in this study reported pain in four or more locations. Participants with PVD reported an average pain intensity of 4.61 (*SD* = 2.00) across all vestibule sites.

### Questionnaires

Trait Subscale of the State-Trait Anxiety Scale (STAI). The STAI (30) is a 40-item measure of self-reported state (present) and trait (stable) anxiety. Greater scores on either 20-item subscale indicate greater feelings of anxiety. The internal consistency of the STAI has been found to be acceptable (30). In the present study, the trait subscale was administered to participants.

The Beck Depression Inventory- II (BDI-II). The BDI-II (31) consists of 21 self-report items assessing depressive symptom severity over the past 4 weeks, with greater scores indicating increased severity. The BDI-II has been shown to have excellent internal reliability and to have convergent validity with measures of depression (32).

### Thermal Pain Testing and Sham MRI

The thermal pain testing and sham MRI sessions were completed at the Queen's MRI Facility Sham Room. The Sham Room is very similar to the actual MRI environment so that participants are able to experience undergoing an MRI, without the magnetic field. Participants were familiarized with the protocol during training on the thermal pain testing protocol and runs in the mock scanner, with the goal of reducing anxiety and providing more consistent functional magnetic resonance imaging (fMRI) results across repeated runs.

Participants were exposed to noxious heat stimulation by resting their right hand on the MRI-compatible robotic contact-heat thermal stimulator (RTS-1; Spinal Map Inc., Kingston). During the thermal pain testing session, a level of temperature for each participant that was considered “moderately painful” was determined on a 101-point scale with verbal descriptors in increments of 10 (i.e., 0 = no sensation, 10 = warm, 20 = a barely painful sensation, 30 = very weak pain, 40 = weak pain, 50 = moderate pain, 60 = slightly strong pain, 70 = strong pain, 80 = very strong pain, 90 = nearly intolerable pain, 100 = intolerable pain). If participants did not rate their pain intensity as “moderately painful” before reaching the safety limit of 51°C, then 51°C was used during trials. A thermode was raised or lowered to contact the thenar eminence (corresponding to the C6 dermatome) through a cut-out in a plexi-glass case, with the timing and temperature controlled by custom-made software on a laptop. The first three contacts lasted for 1.5 s at 45°C with onsets every 3 s. Participants rated the intensity of each individual sensation. This process was repeated with the temperatures of 46 and 47°C after 2 min of rest to avoid sensitization. The goal of this task was to train participants to rate their sensory experience. Following this task, participants rated the intensity of their sensations to 10 consecutive contacts of different temperatures (i.e., 46, 50, 44, and 48*C*), followed by 2 min of rest. Contacts were applied for 1.5 s, with onsets every 3 s, in order to evoke temporal summation of second pain. Repeated applications of brief stimuli can evoke temporal summation of second pain, which is mediated by C-fibers, and provides a robust BOLD response for fMRI studies of pain (25, 33–35). This protocol provided a measure of each participant's pain sensitivity and allowed us to select the appropriate temperature for stimulation during the fMRI session. The thermal pain testing and Sham session lasted ~1 h.

### MRI Acquisition Protocols

Imaging was conducted in a research-dedicated 3 tesla MRI system (Siemens Magnetom Trio). The imaging session took 1.5 h and included one anatomical scan and ten 4.5-min functional scans, guided by initial localizer scans. Participants were positioned with padding and blankets for comfort and to prevent movement. Posterior head, neck, and spine receiver coils were used for detecting the MRI signal, and a mirror was positioned so that participants could view the intensity rating scale and instructions on a rear-projection screen. The peripheral pulse was recorded to account for physiological noise (20, 21).

Localizer images were acquired in three planes for subsequent slice positioning. In order to obtain high quality images with good spatial fidelity, functional MR image data for BS/SC were acquired from the same region as the anatomical scans with a half-fourier single-shot fast spin-echo (HASTE) sequence with 1.5 × 1.5 × 2 mm resolution, and an echo time (TE) of 76 ms (20). Nine sagittal slices spanned from below the disc between the first and second thoracic vertebrae (T1/T2) to above the corpus collosum. This method has been demonstrated to provide optimal T2-weighted BOLD sensitivity and image quality in the spinal cord and brainstem (19–21, 25). HASTE images are less sensitive to field inhomogeneities caused by the anatomy surrounding the spinal cord. It has been shown that the majority of BS/SC fMRI studies to date have utilized variations of single-shot fast spin-echo imaging methods (36). Image data were acquired with a repetition time (TR) of 6.75 s, so each fMRI run consisted of acquiring 40 volumes to describe the BOLD time-series responses.

### fMRI Experimental Design

**Figure 2:**
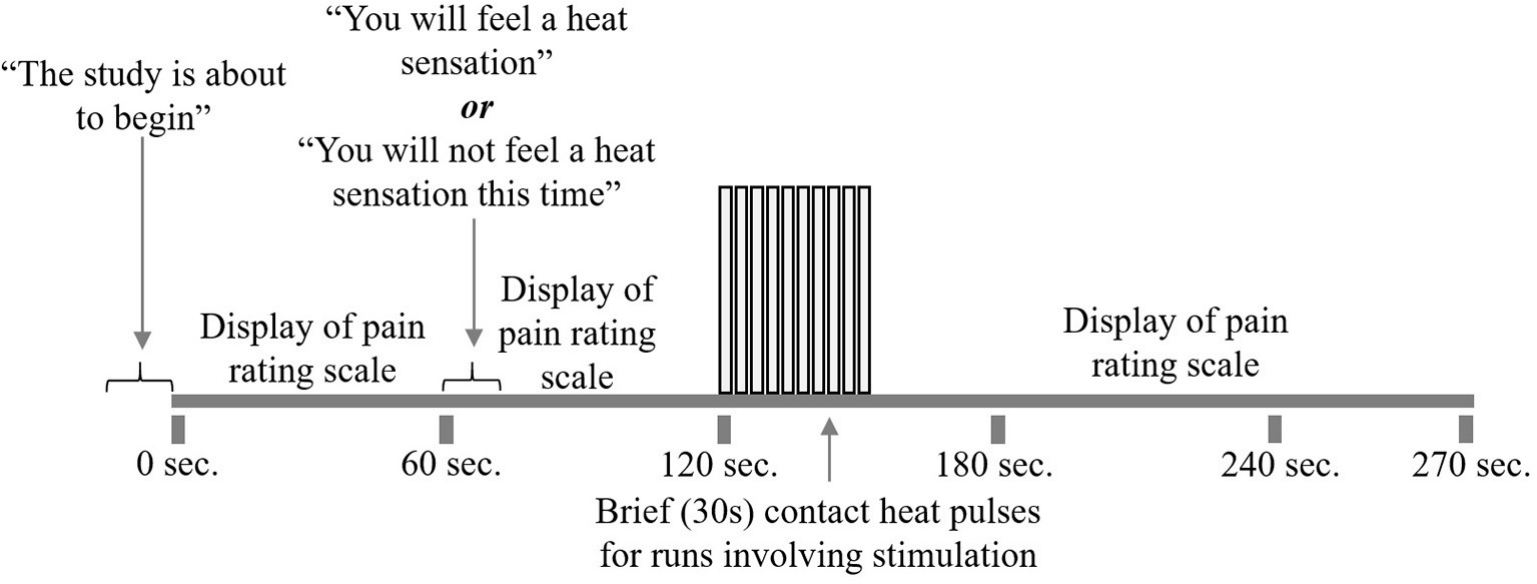
fMRI study design. Participants underwent 10 runs of the study paradigm, five with pain stimulation and five without. First, participants were alerted that the run was about to begin. The pain rating scale was displayed on the screen for their reference. At 1 min, participants were told whether or not to expect heat stimulation for that run. At 2 min, participants began to receive 10 repeated heat contacts of moderately painful temperature, lasting for 30 s. If the run did not include heat stimulation, participants would rest during this 30 s. Following heat stimulation, participants would rest for 2 min while the run and scanning were completed.

At the start of each acquisition participants were informed that a new run was about to begin but they did not initially know if they would receive a painful stimulus (“Pain” condition) or not (“No Pain” condition). One minute after the start of the acquisition, they were informed whether or not they would feel a heat stimulus. For runs without stimulation, data acquisition was continued for a total of 4.5 min, with no stimulus applied. In the “No Pain” condition, participants were instructed to remain still for the duration of the run. If they were to feel a stimulus, it was applied beginning 2 min after the start of the acquisition (i.e., 1 min after they were informed of the stimulus). It consisted of 10 heat contacts applied to the heel of the right thumb, with contact durations of 1.5 s and onsets every 3 s, at the temperature that elicited their moderate level of pain (Figure 2). Pain evoked by repetitive heat stimuli has been shown to result in C-fiber-mediated summation of pain (19). The stimulation period was followed by 2 min without stimulation, totaling 4.5 min of acquisition. Participants were instructed to silently rate their pain for each contact, as practiced during sham training, and at the end of each run were asked to report their ratings for the first and last contacts. If, during the MRI session, participants began to rate their pain as less or greater than moderately painful (either after leaving the sham room or across the MRI session runs), the researcher would titrate the temperature to achieve a moderately painful stimulus. Before beginning the MRI acquisition, after the 5th trial, and following the MRI acquisition participants were asked to verbally “rate your anxiety at this moment” on a 0 (I feel calm and at ease) to 10 (I feel as if I may have an anxiety attack) scale (Table 2). There were a total of five runs for each stimulus type (Pain or No Pain) presented in a pseudo-randomized, counterbalanced order with 2-min breaks to avoid sensitization of the skin.

**Table 2:** Average anxiety ratings before, during, and after MRI sessions.

	**PVD sample**	**Control sample**	***p*-value**
Before MRI [*M* (*SD*)]	1.6 (1.3)	0.9 (1.2)	0.145
	*n* = 14	*n* = 16	
During MRI [*M* (*SD*)]	1.9 (1.3)	0.9 (1.4)	0.056
	*n* = 14	*n* = 16	
After MRI [*M* (*SD*)]	1.7 (2.5)	0.7 (1.2)	0.174
	*n* = 15	*n* = 16	

### Study Procedure

Participants with PVD who passed the initial screening were scheduled for a gynecological examination to confirm their eligibility for inclusion in this study. Women with PVD gave informed consent at the gynecological exam and again at their MRI session, and Control women gave informed consent at their MRI session. They were asked to refrain from alcohol for at least 12 h prior to the imaging portions of the study and from caffeine for at least 6 h before the study, as these substances may affect mood, alertness, and the BOLD response. Participants were also asked to eat a regular meal at their usual mealtime prior to participation in the study.

Before the imaging session, the thermal pain testing/sham MRI session was conducted. Before entering the magnetic imaging environment, participants again confirmed that they had no contraindications for the subsequent magnetic resonance imaging. Participants were asked to change into MRI-safe clothing provided by the facility if any item of clothing possibly contained materials that were not MRI safe. They were then positioned in the MRI for scanning to commence. At the end, participants were debriefed and compensated. This study was part of a larger study that involved a separate session of brain imaging (37), saliva collection, and the administration of validated self-report measures; these additional components are not discussed in this paper.

### Data Processing

The BS/SC data consisted of 5 runs in each of the No Pain and Pain conditions. Pre-processing was conducted with custom-written software in MATLAB called spinalfmri8 (25). First, image data were converted to Neuroimaging Informatics Technology Initiative (NIfTI) format. Sagittal slices were then co-registered to correct for bulk body motion using a 3D, non-rigid registration tool [Medical Image Registration Toolbox; (38, 39)]. These co-registration motion parameters were later used to model bulk movement and reduce noise. Next, the images were spatially normalized to fit an anatomical template previously defined based on 356 healthy participants in previous studies (23, 25). To avoid variability in T1-weighting, the first two volumes were discarded from each time series. Regions of white matter were used to model global noise. Models of noise based on the peripheral pulse, bulk motion parameters, and white matter regions, were fit to the data using a general linear model (GLM) and subtracted from the measured data.

### Structural Equation Modeling (SEM)

**Figure 3:**
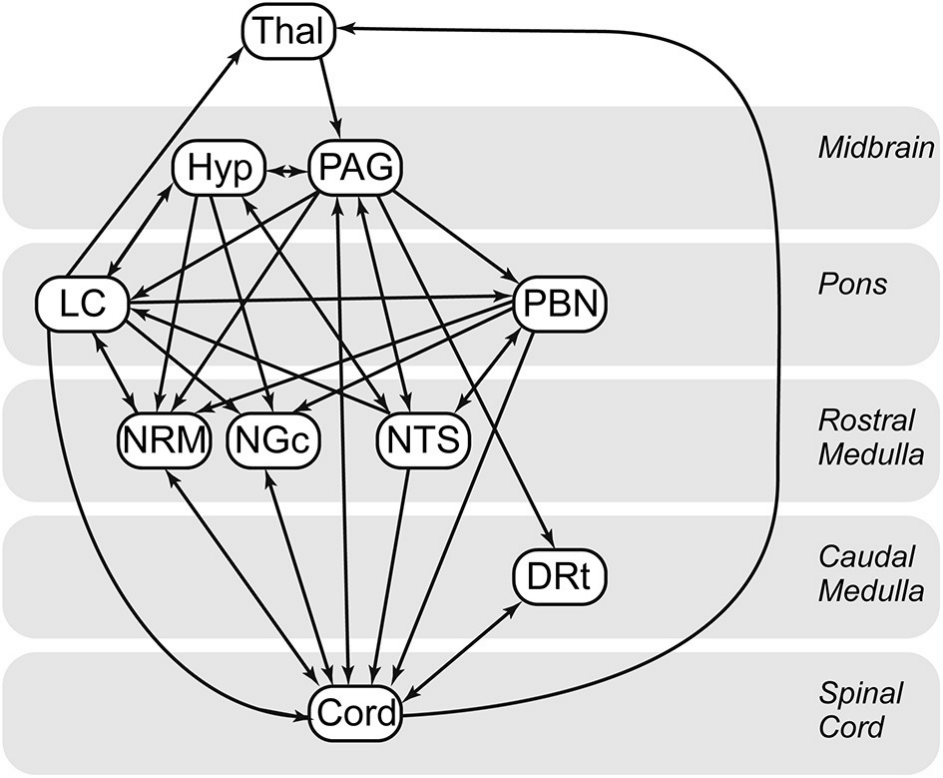
Spinal cord/brainstem SEM anatomical model. Thal, thalamus; Hyp, hypothalamus; DRt, dorsal reticular nucleus; LC, locus coeruleus; NGC, nucleus gigantocellularis; NTS, nucleus tractus solitarius; PAG, periaqueductal gray; PBN, parabrachial nucleus; CORD, C6 right dorsal. Arrows indicate directionality of the connections in the defined model. Single direction arrows indicate a connection that is modeled only in one direction. Arrows in both directions between two regions represent bidirectionality.

Custom-made software written in MATLAB was used to perform SEM analyses for the purposes of assessing functional connectivity. SEM analysis, adapted for our purposes, provides measures of connectivity between regions, based on a pre-defined anatomical model of regions which are known/suspected of playing a role in pain processing, and anatomical connections between regions (40). This method allows us to model multiple sources of input signaling to each region, as opposed to using simple correlations between BOLD responses, which assumes that each region has a single dominant source of input. The additional information provided by an anatomical model also enables the directionality of interactions to be inferred. Based on previous findings of BOLD variations occurring in the spinal cord and brainstem in the periods before and after painful stimulation, and also in the absence of any sensory stimulus, pain has been shown to be continuously modulated by emotional and cognitive factors (23, 41–43). As a result, instead of modeling the influence of continuous cognitive and emotional influences, the approach to analysis used in the present paper is data driven, which has been shown to be appropriate for pain populations (44). The a priori specified model we used (Figure 3) is based on anatomical connections and regions known to be related to pain processing, and includes the thalamus (Thal), hypothalamus (Hyp), periaqueductal gray matter (PAG), locus coeruleus (LC), parabrachial nucleus (PBN), nucleus raphe magnus (NRM), nucleus gigantocellularis (NGc), nucleus tractus solitarius (NTS), dorsal reticular nucleus (DRt), and C6 right dorsal portion of the spinal cord (C6RD) (22, 45–47).

The SEM analysis method consisted of first identifying voxels in the pre-selected regions (based on the anatomical model). K-means clustering was then used to divide each region of interest into seven sub-regions (clusters) based on their BOLD responses to provide finer anatomical precision for identifying connected regions. Voxels were divided and clustered based on functional characteristics, thereby increasing spatial precision. Any clusters which were predominantly along the edge of cerebrospinal fluid (CSF) –filled spaces were removed from the analysis, because these are susceptible to effects of physiological movement. The BOLD time-course responses in each region (as a target for input signaling) was then fit to a linear combination of the BOLD time-course responses in the possible source regions. Region definitions are performed once and are the same across groups and conditions. The linear weighting factors which quantify each region's contribution as an input signal source is termed a β value. Thus, for each target region, a general linear model (GLM) was used, with the source region time-courses as the basis set, and the set of linear weighting factors (β) was calculated. This procedure was repeated for every possible combination of clusters in each source and target region and was repeated for each run and group type. We also allowed the SEM weighting factors to vary dynamically by calculating connectivity strengths using only data from specific epochs, spanning seven volumes, one within the baseline period of a run (75–120 s), one spanning the stimulation period (115–160 s), and one within the baseline period after the stimulation period (153–198 s). Volumes from the selected epoch were concatenated across runs of the same type in each person.

As mentioned above, every combination of sub-regions within each region were investigated to determine the best fit with the BOLD time-series data. The linear weighting factor value for each connection within a network component (i.e., a target cluster and a set of possible source clusters) was thus calculated multiple times with different combinations of source regions included. We calculated the goodness-of-fit by examining the proportion of the variance in the target region that was accounted for by the network component fit (i.e., *R*^2^ value). We determined the probability of each fit occurring by random chance by converting *R*-values to *Z*-scores (Fisher's transform; *Z* = tanh^−1^ (*R*)$\sqrt{tsize-3}$) to estimate the significance of the fit. The fit was repeated by pruning a single source region at a time from the network component to assure all terms accounted for a significant portion of the variance in a target region, using an *F*-test with a corrected cutoff *F*-value corresponding to a *p* < 0.05. If any portion of the network did not account for a significant portion of the variance, it was not included in the results.

The significance of each total network component was determined by previously established probability distributions (40). The distributions depend on model parameters, and the significance thresholds accounted for family wise error rate (*p*_fwe_ < 0.05), taking into consideration the number of network combinations that were tested across the possible combinations of sub-regions. The complete best-fit network was identified for each run type for both PVD and Control women by identifying the combinations of source regions that have the highest *Z*-values for each network component (i.e., the set of clusters with the highest *Z*-scores for each network component). The best-fit model explains the BOLD signal variations in the regions of interest based on known anatomical connections.

Significant differences in connectivity between regions were identified between study conditions using paired sample *t*-tests. Significant differences were inferred between two conditions if the *t*-test culminated in a Bonferroni-corrected *p* < 0.05, accounting for the total number of comparisons. Connections selected for further analysis based on having significant fits (*p*_fwe_ < 0.05) in any of the four combinations of study groups and conditions. Variations in SEM weighting factors between the study groups (PVD or Control), and between conditions (Stimulation or No-Stimulation) were also investigated by means of an analysis of variance (ANOVA). The family-wise error rate correction was applied to the ANOVA. Individual SEM results (β-values) from all participants for a given connection were then analyzed with a 2 × 2 ANOVA with the study group and the study condition as the two independent variables. Results were identified with significant main effects of the group, or condition, or group x condition interactions, at *p* < 0.05.

## Results

### Participant Group Characteristics

Table 1 summarizes the characteristics of the participants in each study group (PVD and Control). Comparisons between groups were made using *t*-tests for continuous variables and Fisher's exact tests for categorical variables (Table 1). We were unable to control for menstrual cycle due to the organization of scheduling within the MRI facility; however, a Fisher's exact tests of estimated menstrual cycle phase (i.e., follicular, ovulatory, luteal, menstrual, or no longer menstruating) based on the start date of participants' most recent menstrual period revealed no significant differences between women with PVD and Control women, *p* = 1.00. The groups only significantly differed in their self-identified ethnicity, with the majority (13/16) of PVD participants identifying as Canadian compared to the Control participants who identified primarily as Canadian (8/16), European (4/16), and Asian (3/16). Due to time constraints, four participants in the PVD group completed the thermal pain testing training session and were familiar with the thermal pain paradigm but were unable to complete the practice runs in the mock scanner. We performed an SEM analysis of BS/SC data excluding these four participants, and there were no differences. Thus, we included all participants in the analyses.

### Thermal Pain Results

Women with PVD reported a wide range of PVD symptom duration, from 1 to 28 years (*M* = 9.1, *SD* = 8.04; based on 10 of the 16 participants who responded to this question). The pain ratings obtained, and temperatures used, during the MRI sessions are summarized in Table 3. These values were averaged over the five trials with stimulation for each participant and then averaged within the study groups. A comparison between groups was done using a *t*-test. Women with PVD required lower temperatures to elicit moderate pain than Control women; however, this difference was not statistically significant.

**Table 3:** Average temperatures and pain ratings during MRI sessions.

	**PVD sample**	**Control sample**	***p*-value**
	***n* = 16**	***n* = 16**	
Average temperature [*M* (*SD*)]	48.9 (2.1)	49.8 (1.1)	0.144
Average pain ratings [*M* (*SD*)]	48.3 (9.5)	49.0 (11.9)	0.685

### BS/SC Group Level SEM Results

Connectivity values across regions were compared between all four participant groups (Control Pain, Control No Pain, PVD Pain, and PVD No Pain) for each of the epochs that were analyzed (before, during, and after stimulation). Results show significant differences in weighting factors (β) between groups, and across time periods, for a number of connections. The significant differences between group and condition comparisons are summarized in Table 4, and the connectivity values for each target/source region pair, and group/condition, are listed. During the stimulation period, there were significant differences in network connectivity between the Control and PVD groups in the Pain condition, and connectivity values tended to be higher in the Control group. These include connections between the PAG and hypothalamus, from the PAG to the NGc, NTS, and DRt, and from the spinal cord to the thalamus. When comparing the No Pain conditions (Control vs. PVD) there were fewer differences between the groups, although differences in connectivity from the PAG to the NTS were found both during what would be the stimulation period, and the period after stimulation. Connectivity values during the No Pain condition tended to be higher in the PVD group.

**Table 4:** Summary of significant differences in spinal cord/brainstem connectivity between all groups, analyzed with SEM.

**Time period**	**Region source → Target**	**Control pain**	**Control no pain**	**PVD pain**	**PVD no pain**
**Control pain vs. Control no pain differences**
Before stimulation	PAG → Hypothalamus	**0.48** **±** **0.08**	**0.11** **±** **0.07**	0.08 ± 0.07	0.18 ± 0.07
	Hypothalamus->PAG	**0.60** **±** **0.08**	**0.19** **±** **0.12**	0.15 ± 0.13	0.34 ± 0.12
During stimulation	PAG → Hypothalamus	**0.58** **±** **0.07**	**−0.00** **±** **0.09**	0.15 ± 0.09	0.13 ± 0.08
	Hypothalamus → PAG	**0.82** **±** **0.05**	**0.00** **±** **0.09**	0.22 ± 0.10	0.21 ± 0.11
	NGc → LC	**0.21** **±** **0.06**	**−0.08** **±** **0.08**	0.02 ± 0.08	−0.15 ± 0.08
	Hypothalamus → NGc	**0.52** **±** **0.10**	**−0.04** **±** **0.12**	−0.07 ± 0.13	0.09 ± 0.15
	PAG → NGc	**0.64** **±** **0.09**	**0.01** **±** **0.13**	0.12 ± 0.12	−0.09 ± 0.12
	PAG → NTS	**0.76** **±** **0.10**	**−0.20** **±** **0.15**	0.14 ± 0.14	0.48 ± 0.16
After stimulation	PAG → NTS	**0.69** **±** **0.06**	**0.07** **±** **0.09**	0.16 ± 0.09	0.17 ± 0.07
	PAG → Hypothalamus	**0.87** **±** **0.07**	**0.11** **±** **0.10**	0.26 ± 0.10	0.30 ± 0.12
	Hypothalamus → PAG	**0.54** **±** **0.09**	**−0.03** **±** **0.12**	0.32 ± 0.11	0.01 ± 0.11
	PAG → NGc	**0.26** **±** **0.08**	**−0.31** **±** **0.16**	0.05 ± 0.16	0.40 ± 0.13
	PAG → NTS	**0.26** **±** **0.08**	**−0.31** **±** **0.16**	0.05 ± 0.16	0.40 ± 0.13
	PAG → DRt	**0.28** **±** **0.04**	**−0.02** **±** **0.10**	0.08 ± 0.11	0.20 ± 0.12
**PVD pain vs. PVD no pain**
During stimulation	C6 RD → Thalamus	0.36 ± 0.07	0.38 ± 0.07	**0.02** **±** **0.07**	**0.33** **±** **0.07**
**Control no pain vs. PVD no pain**
During stimulation	PAG → NTS	0.76 ± 0.10	**−0.20** **±** **0.15**	0.14 ± 0.14	**0.48** **±** **0.16**
After stimulation	PAG → NTS	0.26 ± 0.08	**−0.31** **±** **0.16**	0.05 ± 0.16	**0.40** **±** **0.13**
**Control pain vs. PVD pain**
Before stimulation	PAG → Hypothalamus	**0.48** **±** **0.08**	0.11 ± 0.07	**0.08** **±** **0.07**	0.18 ± 0.07
	Hypothalamus → PAG	**0.60** **±** **0.08**	0.19 ± 0.12	**0.15** **±** **0.13**	0.34 ± 0.12
During stimulation	C6 RD → Thalamus	**0.36** **±** **0.07**	0.38 ± 0.07	**0.02** **±** **0.07**	0.33 ± 0.07
	PAG → Hypothalamus	**0.58** **±** **0.07**	−0.00 ± 0.09	**0.15** **±** **0.09**	0.13 ± 0.08
	Hypothalamus → PAG	**0.82** **±** **0.05**	0.00 ± 0.09	**0.22** **±** **0.10**	0.21 ± 0.11
	PAG → PBN	**0.26** **±** **0.03**	0.13 ± 0.10	**−0.03** **±** **0.09**	−0.02 ± 0.09
	Hypothalamus → NGC	**0.52** **±** **0.10**	−0.04 ± 0.12	**−0.07** **±** **0.13**	0.09 ± 0.15
	PAG → NGc	**0.64** **±** **0.09**	0.01 ± 0.13	**0.12** **±** **0.12**	−0.09 ± 0.12
	PAG → NTS	**0.76** **±** **0.10**	−0.20 ± 0.15	**0.14** **±** **0.14**	0.48 ± 0.16
	PAG → DRt	**0.35** **±** **0.04**	0.16 ± 0.12	**−0.03** **±** **0.10**	0.27 ± 0.11
After stimulation	PAG → Hypothalamus	**0.69** **±** **0.06**	0.07 ± 0.09	**0.16** **±** **0.09**	0.17 ± 0.07
	Hypothalamus → PAG	**0.87** **±** **0.07**	0.11 ± 0.10	**0.26** **±** **0.10**	0.30 ± 0.12
	PAG → PBN	**0.22** **±** **0.03**	0.17 ± 0.09	**−0.07** **±** **0.09**	−0.03 ± 0.10
	Hypothalamus → NGC	**0.46** **±** **0.11**	0.12 ± 0.12	**−0.09** **±** **0.12**	0.29 ± 0.15
	PBN → NGc	**0.41** **±** **0.11**	0.06 ± 0.12	**−0.08** **±** **0.12**	−0.03 ± 0.10
	Hypothalamus → NTS	**0.48** **±** **0.10**	0.21 ± 0.14	**0.00** **±** **0.13**	0.14 ± 0.12
	C6 RD → DRt	0.34 ± 0.08	0.20 ± 0.07	−0.00 ± 0.07	−0.02 ± 0.07

*Columns contain the weighting factor (β) of the specific connection and the respective error, while bolded pairs in the columns denote which pairs of β values are significantly different. Only connections with significant differences in at least one comparison of group/condition are shown here. DRt, dorsal reticular nucleus; LC, locus coeruleus; NRM, Nucleus Raphe Magnus; NGc, nucleus gigantocellularis; NTS, nucleus tractus solitarius; PAG, periaqueductal gray; PBN, parabrachial nucleus*.

In the Control group, comparing Pain and No Pain conditions, higher connection strengths were consistently observed in the Pain condition. This included in the period before stimulation in PAG-hypothalamus connectivity and during the stimulation period in connectivity between the PAG and hypothalamus, NGc to LC, hypothalamus to NGc, and PAG to both NGc and NTS regions. In contrast, comparing the Pain and No Pain conditions in the PVD group revealed differences only in the spinal cord to thalamus connectivity, during the stimulation period, and the connectivity strength was stronger in the No Pain condition.

### ANOVA Results

**Figure 4:**
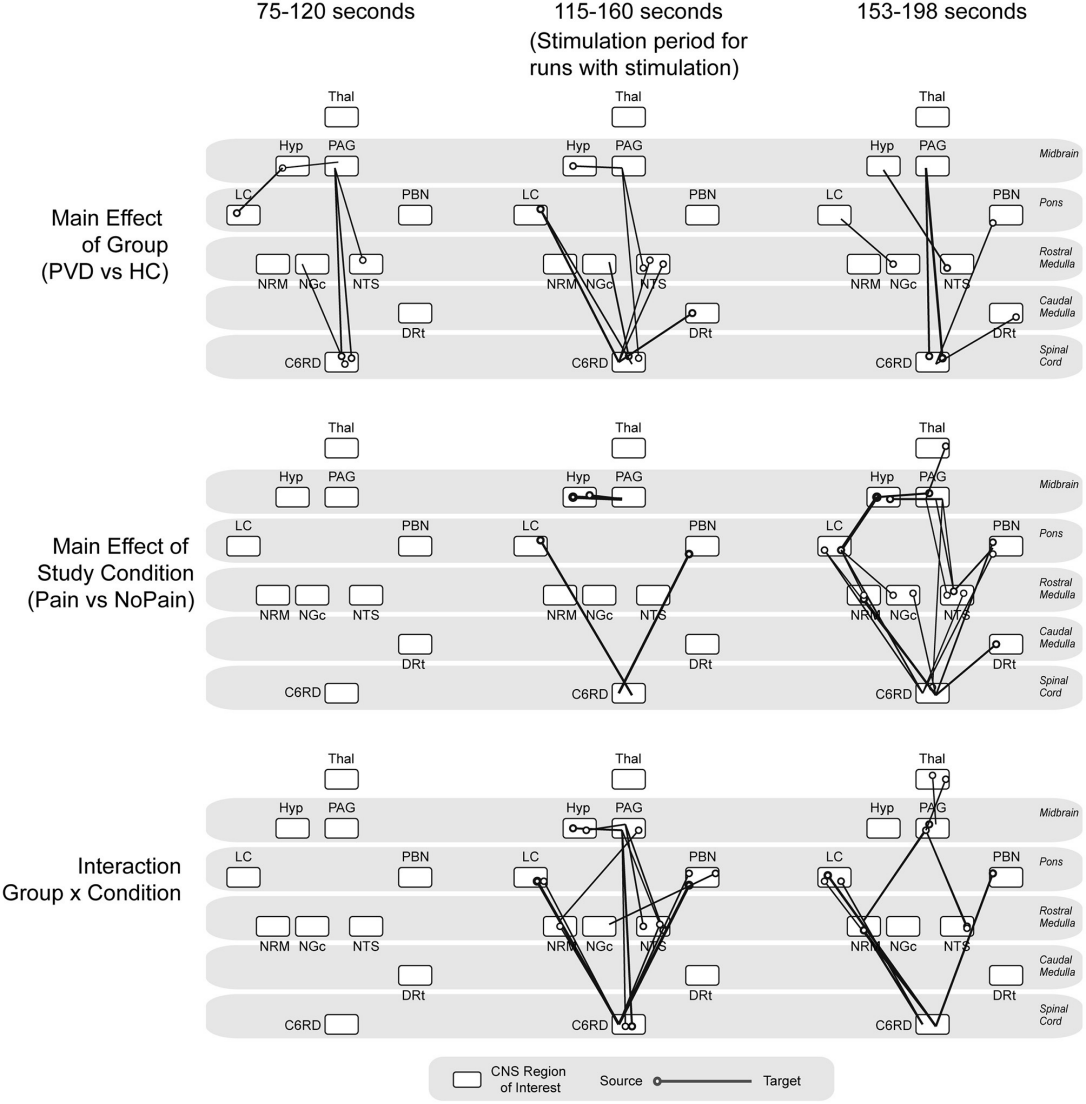
ANOVA results. Summarizing main effects of group (Control vs. PVD), study condition (pain vs. no pain) and group × condition interaction effects, analyzed at three different time points (baseline period before stimulation would be applied, during noxious stimulation, baseline period after the stimulation as ended). The circles indicate the source for each connection and the line is drawn to the target region.

The analysis of variances (ANOVAs) of connectivity strengths in the SC and BS networks in relation to the group (Control participants vs. women with PVD) and the condition (Pain vs. No Pain), as well as the group × condition interaction effects, are shown in Figure 4. The ANOVA results identified several connections for which connectivity strengths varied specifically with the group (Control or PVD), regardless of the stimulation condition (Pain or No Pain applied) in all three time periods that were analyzed (before the painful stimulus would be applied, during painful stimulation, and after the stimulus was applied). These involved connections to and from the hypothalamus, PAG, LC, NRM, NGc, NTS, DRt, and PBN regions. Additionally, during the baseline period before a stimulus would be applied, ANOVA results showed connections that varied specifically with the group (Control vs. PVD), namely from the LC to the hypothalamus, from the hypothalamus and NTS to the PAG, and from the spinal cord to the NGc and PAG, but no connections exhibited significant condition or interaction effects.

During the stimulation period, there was a main effect of study condition (Pain vs. No Pain) in connectivity from the LC and PBN to the spinal cord, and from the hypothalamus to the PAG. The connectivity strengths of several connections such as from the LC, NTS, and DRt to the spinal cord, from the hypothalamus to the PAG, from the PAG to the NTS, and from spinal cord to the NRM, NGc, and PAG also varied significantly between the Control and PVD groups. However, during this time period, connectivity strengths varied mostly with group × condition interaction effects, primarily in connections from the LC and PBN to the spinal cord, the hypothalamus to the PAG, and from the PAG and the NRM to the spinal cord.

## Discussion

This study is the first to examine BS/SC connectivity during Pain and No Pain conditions in participants with and without PVD. The aim of this study was to investigate differences in descending modulatory mechanisms in women with PVD as compared to Control participants. A key feature of our results is that women with PVD were observed to have similar connectivity values between regions of the BS/SC known to play a role in pain perception regardless of whether or not a noxious heat stimulus was applied to their hand, suggesting they consistently exhibited altered pain processing on a neurophysiological level, in contrast to Control participants without chronic pain.

During the application of a noxious heat stimulus to the hand, the SEM analysis identified differences between the Control and PVD groups in regions related to descending modulation of pain. These include connections from the PAG to the hypothalamus, PBN, NGc, NTS, and DRt, the spinal cord to the thalamus, and the hypothalamus to the PAG and NGC. Similar to the PAG, the hypothalamus has been found to play an integral role in pain processing, and stimulation of the hypothalamus can inhibit the response of neurons in the spinal cord to painful stimuli (47). There are antinociceptive pathways known to exist between the hypothalamus and PAG and structures important to affective pain processing, such as the pre-frontal cortex (47). Based on the ANOVA results, the PVD and Control groups showed significant differences in connectivity strengths between a number of regions (i.e., LC, NTS, and DRt to the spinal cord, the hypothalamus to the PAG, the PAG to the NTS, and the spinal cord to the NRM, NGc, and PAG). Significant interaction effects were also identified during pain stimulation (i.e., LC and PBN regions to the spinal cord, hypothalamus to PAG, and PAG to NRM and spinal cord). Based on these findings, we conclude that women with PVD have altered pain processing at the level of the BS and SC compared to Control participants.

There were fewer differences between the two groups during the No Pain condition than during the Pain condition. However, there were interesting connectivity differences during the stimulation period (i.e., the period when pain would be expected) of the No Pain condition between the PAG and NTS. The presence of differences even when noxious stimulation was not applied indicates that women with PVD exhibit altered pain processing at all times, not only when pain is provoked. This finding indicates that there are differences in the cognitive/emotional state between the groups and in how this state influences neural signaling between regions involved with pain processing in the brainstem and spinal cord. Previous research using resting-state fMRI to investigate differences in intrinsic connectivity in networks related to pain processing found that women with PVD displayed alterations of intrinsic connectivity in networks relevant to sensorimotor function and salience in comparison to Control participants (14). Although the current study did not investigate the BOLD response during a true resting-state, these results show that women with PVD exhibit changes in pain processing during the no-stimulation condition. This effect may be due to altered descending regulatory mechanisms that are related to the vulvar hypersensitivity and allodynia observed in the PVD participants that we studied. When comparing study conditions within the PVD group, the only evident difference was from the spinal cord to the thalamus during the stimulation period. This is in contrast to the Control participants who exhibited significant differences between conditions before stimulation (PAG-Hyp) and during stimulation (PAG to Hyp, NGc to LC, Hyp to NGC, and PAG to both NGC and NTS). Significant differences in connectivity between Pain and No Pain conditions is consistent with previous studies of Control participants and is therefore notably absent in women with PVD in the present study (23, 42). We can only speculate whether differences found between groups for the No-Pain condition during the period when a stimulus would be applied in the Pain condition could reflect relief that there was no stimulus, or uncertainty as to whether a stimulus would actually be applied.

There is limited previous research examining brainstem connectivity in clinical pain populations. However, if chronic pain conditions such as PVD and fibromyalgia have similar features in terms of altered descending pain regulation, these similarities may hint at common elements of these conditions. A study examining connectivity in women with fibromyalgia found that Control participants displayed significantly stronger connectivity from the anterior cingulate cortex (ACC) to multiple brain regions (including the brainstem) and had significantly stronger connectivity from the thalamus to the orbitofrontal cortex. Patients with fibromyalgia also consistently exhibited greater connectivity than Control participants (48). In addition, an examination of the nucleus accumbens in those with Chronic Low Back (CLB) pain in response to the expectation and receipt of brief painful stimuli found similar responses between participants with CLB and Control participants during the onset of the painful stimulus (49, 50). However, Control participants showed increased activity during the falling phase of pain stimulation (i.e., the “offset” or drop in pain stimulation), unlike CLB participants who exhibited reduced activity. Further examination of these findings suggested that the nucleus accumbens response during the falling phase of stimulation was positively associated with the intensity of pain stimulation for Control participants in contrast to the negative relationship for CLB participants, suggesting the nucleus accumbens responds to falling pain stimulation differently based on the context of the participant (Control participant or experiencing persistent pain) (49, 50). The lack of a difference in responses between Pain and No Pain conditions in women with PVD indicates an insufficient response of the pain modulation system, therefore reducing identifiable differences between conditions. This could be the result of it being constantly engaged, and so a further augmented response cannot be observed, or due to dysfunction resulting in the system not becoming engaged when needed. In the accompanying investigation of responses in cortical regions, differences between Pain and No Pain conditions were observed in PVD before, during, and after stimulation, but were observed in Control participants only during stimulation. This observation supports the conclusion that descending pain regulating systems may be constantly engaged in PVD.

Our results provide strong evidence that women with PVD consistently exhibit altered pain processing on a neurophysiological level, in contrast to Control participants. More research is required to explore these findings and elucidate the underlying causes of altered pain in women with PVD.

### Limitations

When performing SEM, we chose regions of interest based on previous research of descending pain regulation for connectivity analyses. As a result, there could be regions relevant to pain processing that were not included in the network model. However, by including more regions to ensure all possible anatomical structures are accounted for, we would increase the number of comparisons performed and run the risk of including non-significant network connections that weaken the strength of β values for each network component. In addition, PVD is a heterogeneous patient group with a wide range of symptom severity, comorbidities, and psychosocial characteristics. It is likely that many of these factors could contribute to alterations in pain processing mechanisms, especially with the knowledge that emotional states play a significant role in pain perception, which were not controlled for in the present analyses (51). However, women with and without PVD did not significantly differ on trait anxiety or severity of depressive symptoms (Table 1), and they did not significantly differ on self-reported anxiety measured on a 0–10 scale before, during, and after the MRI acquisition (Table 2).

### Implications and Conclusions

This study is the first to examine the BS/SC of women with PVD, and it advances our understanding of altered central processing in this condition. The results demonstrate alterations in connectivity between BS/SC regions that are relevant to pain modulation and suggest that descending pain regulatory systems play a role in the expression of PVD. In comparison to Control participants, women with PVD displayed fewer differences in connectivity between stimulation conditions, indicating an insufficient response of the pain modulation system in women with PVD. In addition, it appears that women with PVD exhibit alterations in pain processing even in the absence of provoked pain. Our results provide strong evidence that women with PVD consistently exhibit altered pain processing on a neurophysiological level, a pattern also demonstrated in an investigation of brain connectivity in women with and without PVD (37). Further research is needed to investigate whether constant engagement of descending pain regulation is a common feature of chronic pain conditions.

## Data Availability Statement

The raw data supporting the conclusions of this article will be made available by the authors, without undue reservation.

## Ethics Statement

The studies involving human participants were reviewed and approved by Queen's University Health Sciences and Affiliated Teaching Hospitals Research Ethics Board (HSREB). The patients/participants provided their written informed consent to participate in this study.

## Author Contributions

LY and PS: conceptualization, data acquisition, formal analysis, methodology, writing-original draft, and writing-review and editing. CP: conceptualization, funding acquisition, data acquisition, methodology, re-sources, supervision, writing-original draft, and writing-review and editing. GI: data acquisition, formal analysis, methodology, and writing-review and editing. SC: conceptualization, data acquisition, and writing-review and editing. All authors contributed to the article and approved the submitted version.

## Conflict of Interest

The authors declare that the research was conducted in the absence of any commercial or financial relationships that could be construed as a potential conflict of interest.

## Publisher's Note

All claims expressed in this article are solely those of the authors and do not necessarily represent those of their affiliated organizations, or those of the publisher, the editors and the reviewers. Any product that may be evaluated in this article, or claim that may be made by its manufacturer, is not guaranteed or endorsed by the publisher.
